# Supplementary material for: Aging-regulated anti-apoptotic long non-coding RNA Sarrah augments recovery from acute myocardial infarction
Source: Nat Commun. 2020 Apr 27;11:2039. doi: 10.1038/s41467-020-15995-2 (PMC7184724; doi:10.1038/s41467-020-15995-2)
Supplement: Supplementary file 1 — Supplementary Information [file 41467_2020_15995_MOESM1_ESM.pdf]

Supplementary Materials

Aging-Regulated Anti-Apoptotic Long Non-Coding RNA *Sarrah* Augments Recovery from Acute Myocardial Infarction.

Trembinski et al.

Supplementary Figure 1

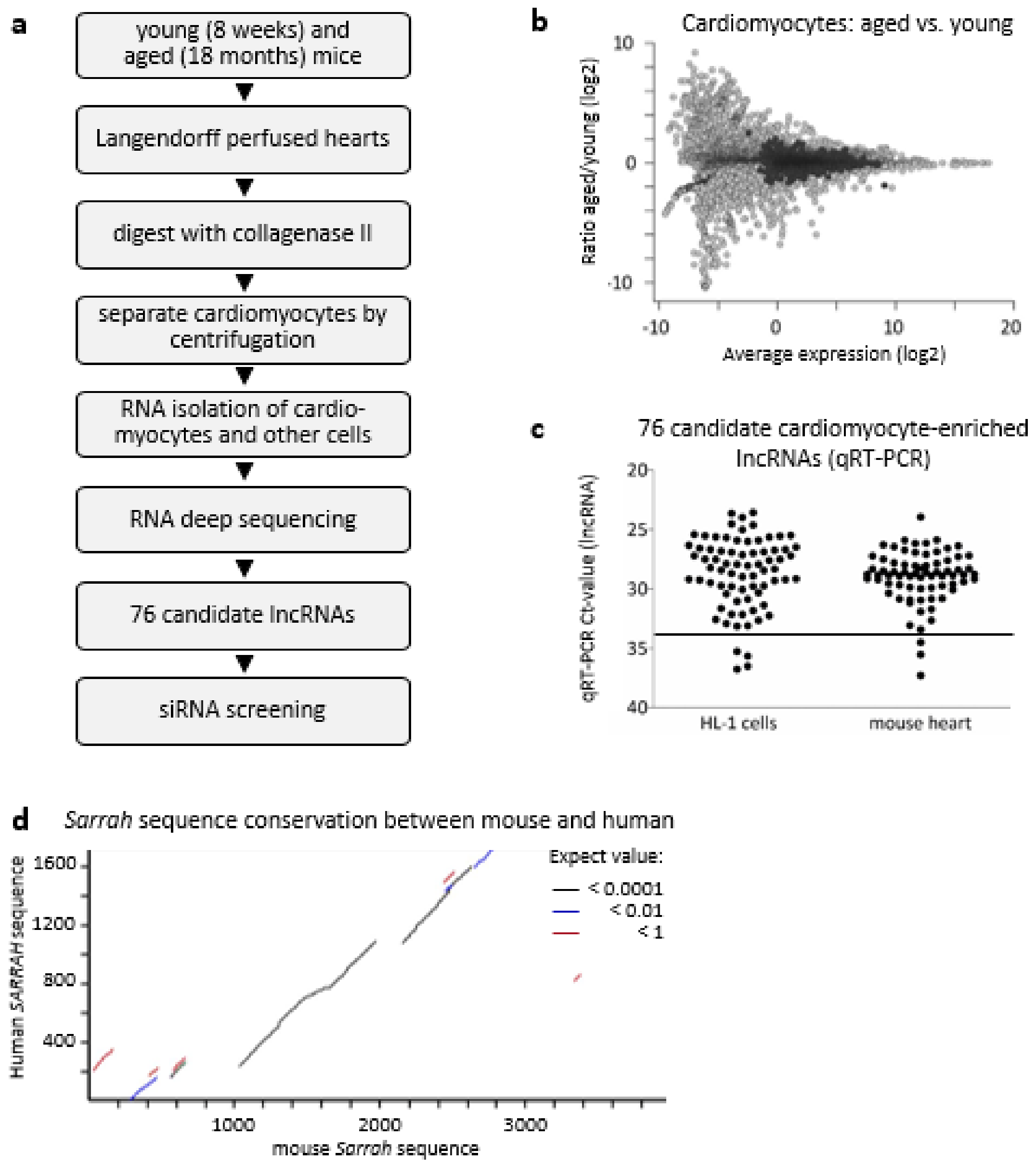

Supplementary figure 1 Several lncRNAs are enriched in cardiomyocytes and regulated during aging.

- **a.** Flow chart illustrating the procedure of primary mouse cardiomyocyte RNA deep sequencing and lncRNA identification.
- **b.** Polyadenylated RNA of cardiomyocytes and remaining heart cells isolated from 8 weeks and 18 months old mice, respectively, was sequenced on the Illumina Hiseq platform (n = 3). Coding transcripts are depicted in light gray, non-coding RNA transcripts in dark gray.
- **c.** Expression of 76 cardiomyocyte-enriched lncRNAs from **(b)** was confirmed by qRT-PCR (cut-off: Ct-value ≤ 34) in HL-1 cells and adult mouse cardiac tissue (n = 3).
- **d.** Sequence conservation between human and mouse *Sarrah* was assessed using the LALIGN DNA:DNA tool of the FASTA Sequence Comparison software of University of Virginia.

Supplementary Figure 2

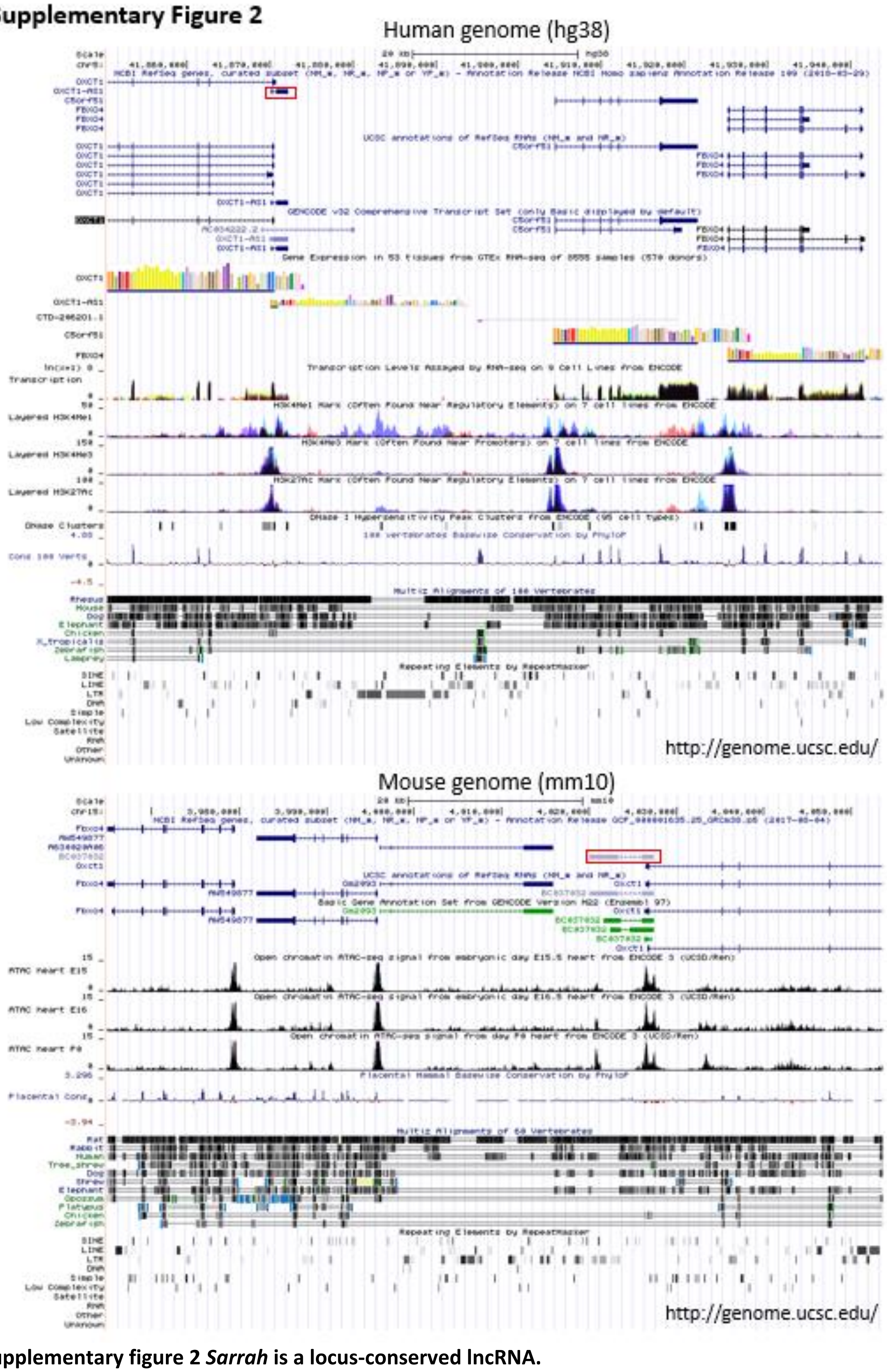

Supplementary figure 2 *Sarrah* is a locus-conserved lncRNA.

Images from the UCSC genome browser showing the locus of *SARRAH/Sarrah*. The annotated *SARRAH/Sarrah* transcript is depicted in a red box.

Supplementary Figure 3

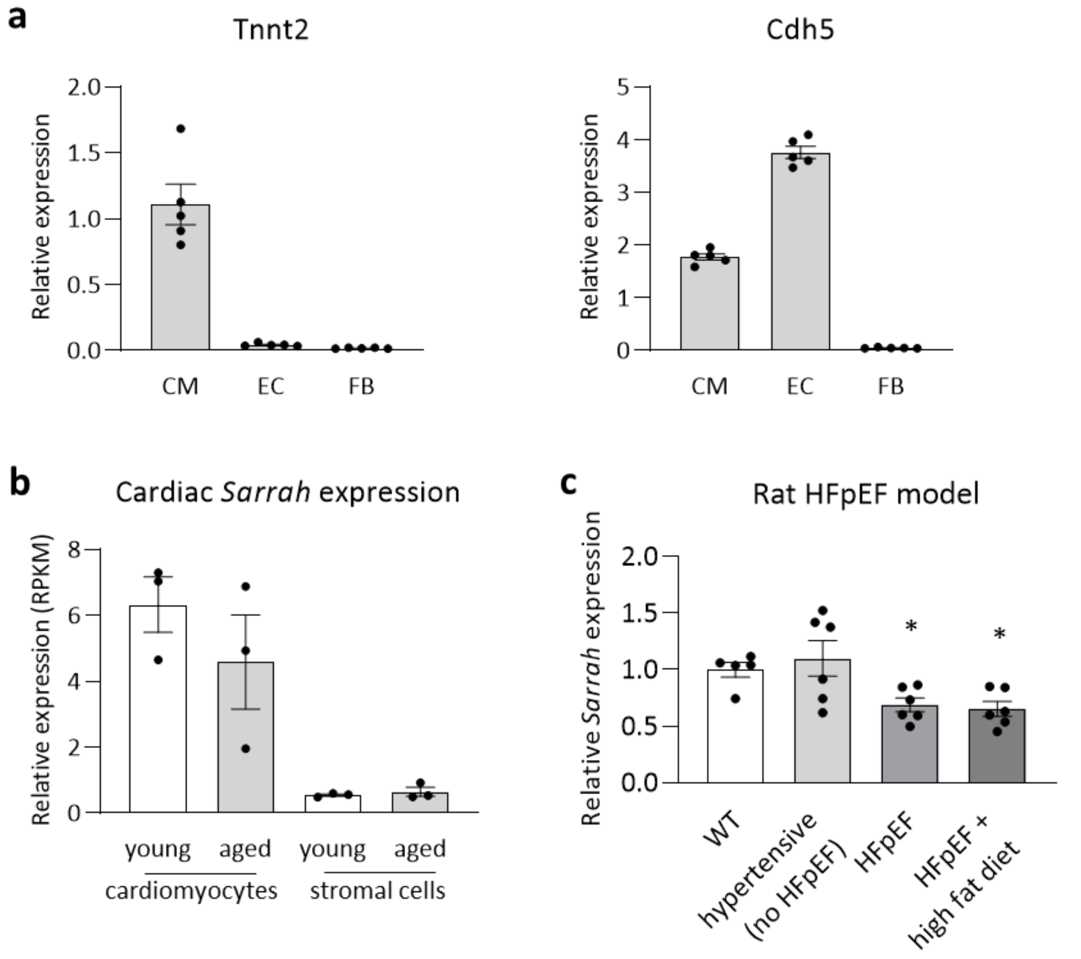

Supplementary figure 3 Sarrah expression in heart cells and a rat HFpEF model

a. RNA was isolated from mouse heart cell fractions from figure 1C (cardiomyocytes (CM), endothelial cells (EC) and fibroblasts (FB)) and levels of cell type-specific markers (Tnnt2 for CM, Cdh5 for EC) were determined by qRT-PCR (n = 5; SEM).

b. Different cell fractions (cardiomyocytes and stromal cells) isolated from young and aged mice were used for RNA isolation. The cardiac *Sarrah* expression profile was determined by RNA deep sequencing (n = 3; SEM).

c. *Sarrah* levels were measured in rat hearts without (wildtype (WT); hypertensive) and with (obese; obese + high fat diet (HFD)) heart failure with preserved ejection fraction (HFpEF) phenotype (n = 5 and 6; SEM; * one-way ANOVA, F = 5.3, p = 0.0202 for HFpEF, p = 0.0137 for HFpEF + High fat diet).

Supplementary Figure 4

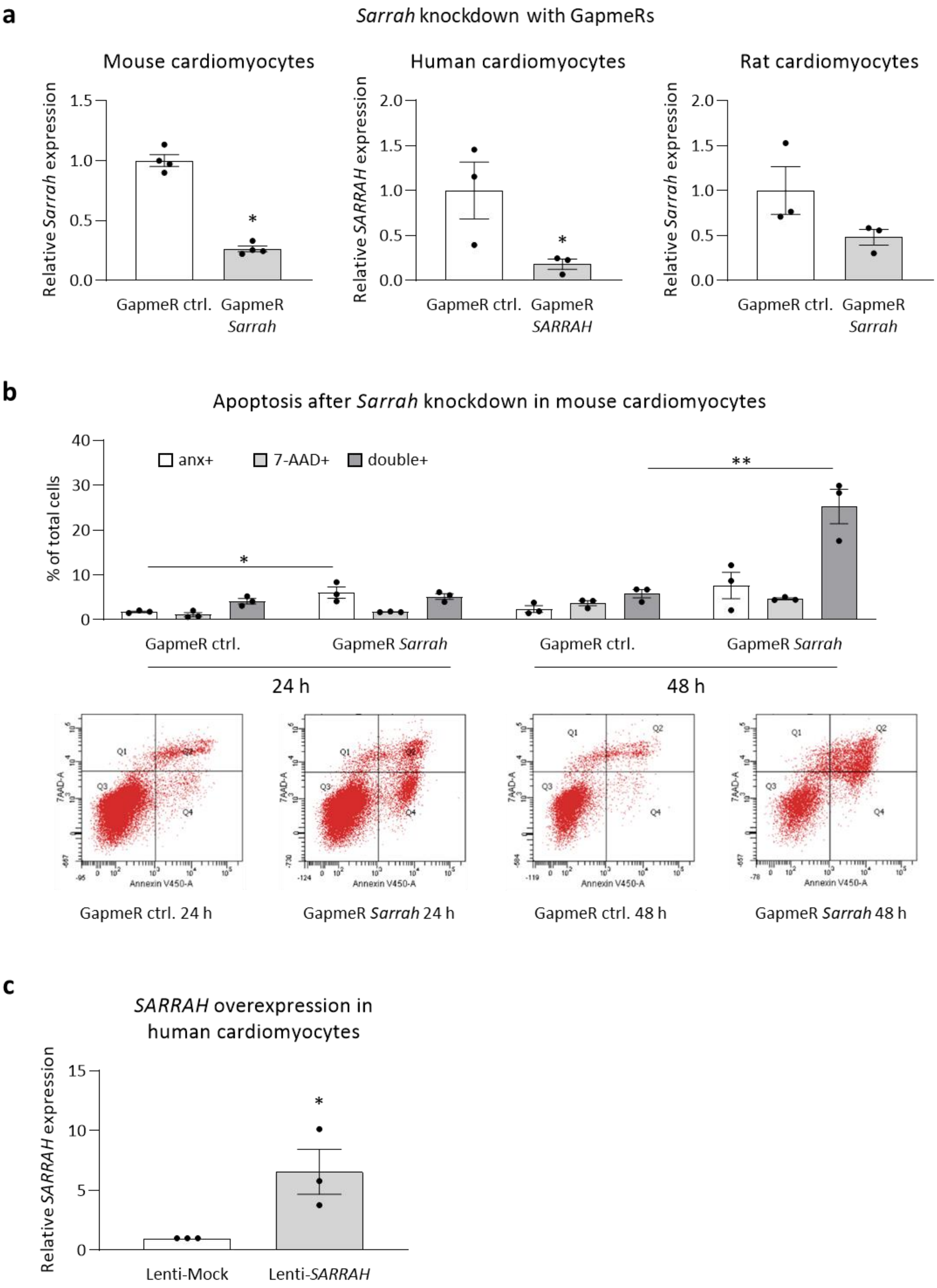

Supplementary figure 4 *Sarrah* silencing induces apoptosis.

a. LNA-DNA-based antisense oligonucleotides that induce RNase H-mediated cleavage of the targeted RNA in the nucleus, so-called GapmeRs, were transfected in mouse (HL-1 cells line), human (primary) and rat (primary) cardiomyocytes. *Sarrah* levels were measured by qRT-PCR (n = 3 and 4; SEM; * t-test p = 0.029 for mouse cardiomyocytes, p = 0.003 for human cardiomyocytes).

b. *Sarrah*-silenced mouse cardiomyocytes (HL-1 cell line) were used for Annexin V/7-AAD stainings. The cells were stained with annexin V-450 and 7-AAD and subsequently analyzed by flow cytometry 24 h or 48 h after transfection. While annexin V specifically stains apoptotic cells, 7-AAD stains all permeable cells, regardless whether necrotic or apoptotic. Early apoptosis manifests as annexin V single positive cells, late apoptosis as double positive cells. The percentage of stained cells is shown as a mean of three independent experiments after 24 h and 48 h, respectively (n = 3; SEM; * two-way ANOVA, F = 13.6 for variable gapmeR treatment, p = 0.0018; ** two-way ANOVA, F = 26.0 for variable gapmeR treatment, p < 0.0001).

c. Primary human cardiomyocytes were transduced with lentiviral vectors. *SARRAH* overexpression was assessed by qRT-PCR (n = 3; SEM; * t-test p = 0.0245).

Supplementary Figure 5

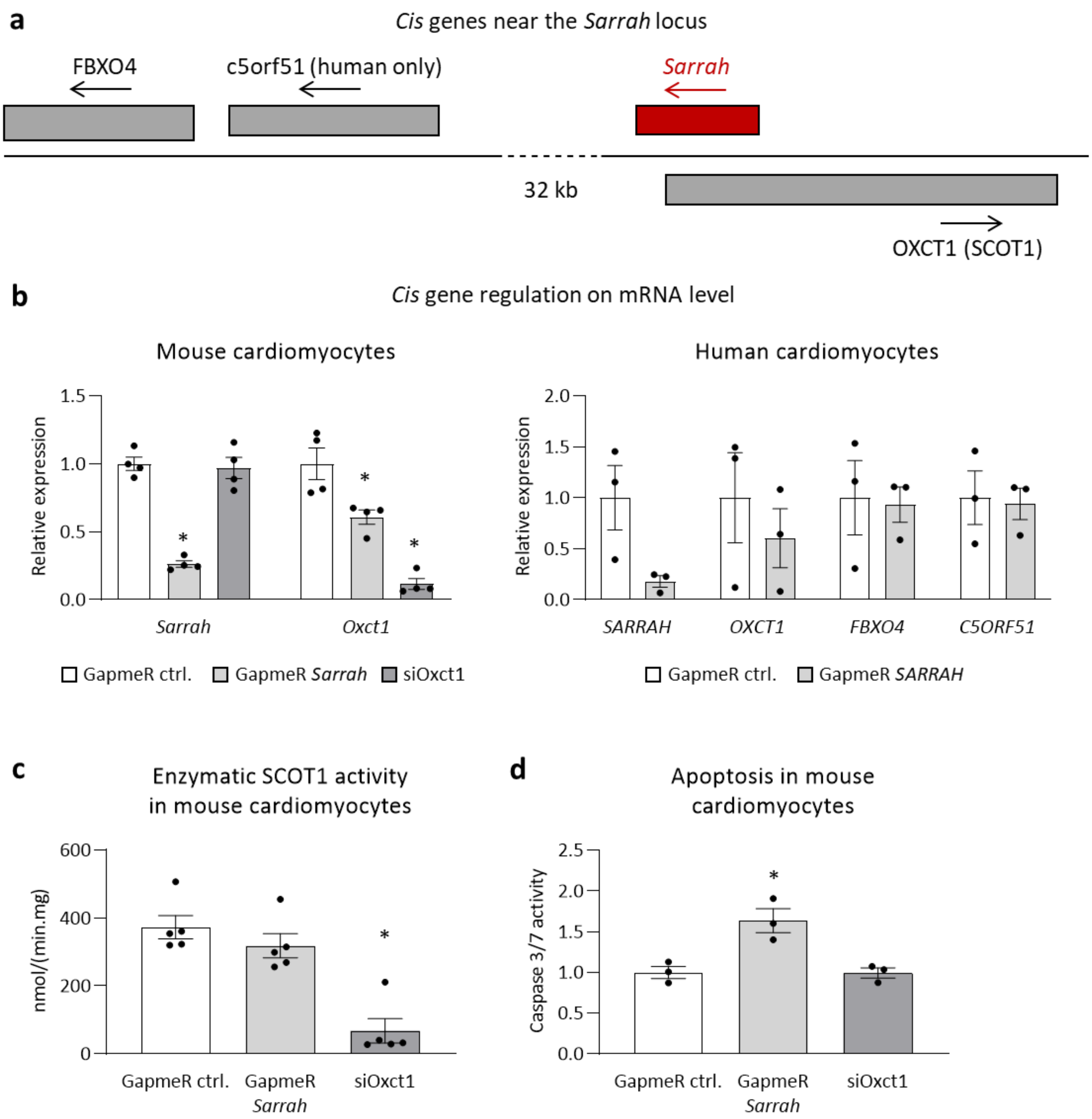

Supplementary figure 5 *Sarrah* does not regulate *cis* genes.

a. Scheme depicting the *Sarrah* locus and neighboring genes.

b. The effects of *Sarrah* silencing on *cis* gene mRNA levels were measured by qRT-PCR in mouse (HL-1 cell line; left; *cis* genes: Oxct1, Fbxo4) and primary human (right, *cis* genes: OXCT1, FBXO4, C5ORF51) cardiomyocytes (n = 3 and 4; SEM; * two-way ANOVA, F = 40.4 for variable treatment, p < 0.0001 for Sarrah after gapmeR Sarrah treatment, p = 0.0012 for Oxct1 after gapmeR Sarrah treatment, p < 0.0001 for Oxct1 after siOxct1 treatment). siRNA knockdown of Oxct1 was performed as a positive control for the regulation of Oxct1 mRNA levels.

c. The effects of *Sarrah* silencing on SCOT1 enzymatic activity were measured by an enzymatic activity assay in mouse cardiomyocytes (HL-1 cell line; n = 5; SEM; * one-way ANOVA, F =21.1, p = 0.0001). siRNA knockdown of OXCT1 was performed as a positive control for the regulation of SCOT1 enzymatic activity.

d. The effects of OXCT1 silencing on apoptosis were measured by a caspase-3/7 activity assay in mouse cardiomyocytes (HL-1 cell line; n = 3; SEM; * one-way ANOVA, F = 13.1 p = 0.0081).

Supplementary Figure 6

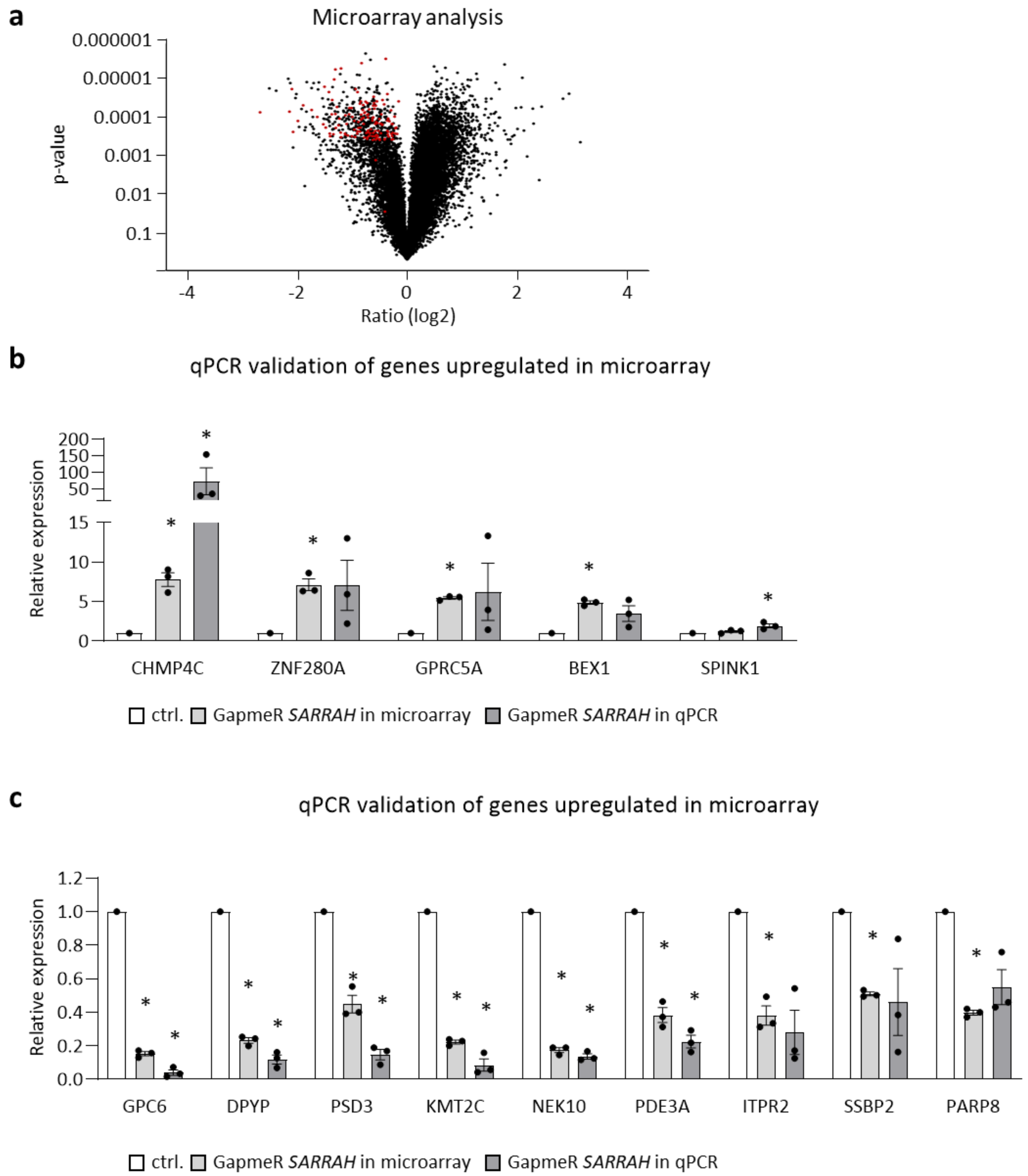

Supplementary figure 6 *SARRAH* has profound effects on gene expression.

a. A microarray analysis was performed with GapmeR-treated primary human cardiomyocytes (GapmeR ctrl. vs. GapmeR *SARRAH*; n = 3). A volcano plot shows all genes measured in the microarray that are down- or upregulated more than -2-fold or 2-fold, respectively. Genes that have predicted *SARRAH* binding sites in their promoters (*SARRAH* target genes) are highlighted in red.

b. Regulation of some of the most significantly upregulated genes from the microarray was confirmed by qRT-PCR (n = 3; SEM; * t-test, p = 0.0032, 0.0027, 0.0003, 0.0009 for CHMP4C, ZNF280A, GPRC5 and BEX1 microarray, respectively. P = 0.0169 for CHMP4C qPCR and p = 0.0397 for SPINK1 qPCR).

c. Regulation of some of the most significantly downregulated genes from the microarray and of some of the predicted *SARRAH* target genes that are involved in apoptosis was confirmed by qPCR (n = 3; SEM; * t-test p = 0.0020, 0.0025, 0.0185, 0.0012, 0.0025, 0.0135, 0.0200, 0.0011 and 0.0015 for GPC6, DPYP, PSD3, KMT2C, NEK10, PDE3A, ITPR2, SSBP2 and PARP8 microarray, respectively. P = 0.0160, 0.0133, 0.0147, 0.0224, 0.0029, and 0.0120 for GPC6, DPYP, PSD3, KMT2C, NEK10 and PDE3A qPCR, respectively).

Supplementary Figure 7

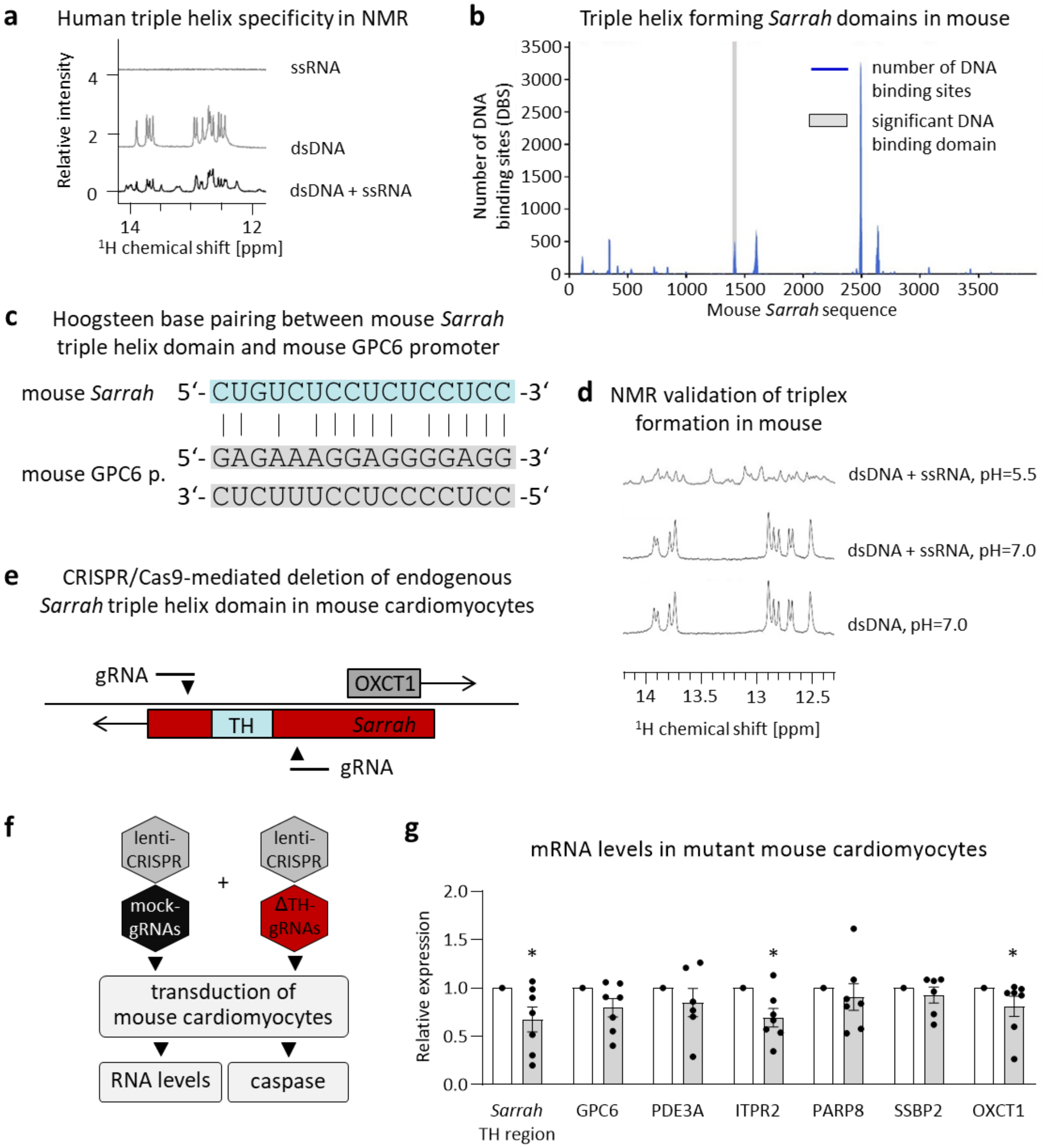

Supplementary figure 7 Triple helix formation between *Sarrah* and gene promoters.

a. ^{1}H spectra of human sequences of *SARRAH* triple helix region and GPC promoter binding site were analyzed as ssRNA (top), dsDNA (middle) and dsDNA + ssRNA (bottom) by nuclear magnetic resonance (NMR). Conditions were 100 µM nucleic acid, 10 mM $NaPO_4$, 50 mM NaCl, 3 mM EDTA, pH = 5.5 at 288 K.

b. The mouse *Sarrah* sequence was assessed with regard to pyrimidine-rich regions capable of DNA binding via triple helix formation using the Triplex Domain Finder software.

c. Scheme indicating Hoogsteen base pairing between the mouse *Sarrah* triple helix domain and the mouse GPC6 promoter.

d. ^{1}H spectra of mouse sequences of *Sarrah* triple helix region and GPC promoter binding site were analyzed as dsDNA only and dsDNA together with ssRNA at both neutral and acidic pH by nuclear magnetic resonance (NMR). Conditions were 100 µM nucleic acid, 10 mM $NaPO_4$, 50 mM NaCl, 3 mM EDTA at 288 K.

e. Scheme showing a CRISPR/Cas9-mediated approach to delete the endogenous *Sarrah* triple helix domain (TH) from mouse cardiomyocytes (HL-1 cell line).

f. Flow chart illustrating the generation and experimental use of mouse cardiomyocytes (HL-1 cell line) lacking the endogenous triple helix-forming domain. Cells were simultaneously transduced with CRISPR/Cas9- and gRNA-containig viruses and used for downstream applications two days after tranduction.

g. Levels of *Sarrah* triple helix-containing transcript, *Sarrah* target genes and OXCT1 in mock-transduced and *Sarrah* triple helix domain-excised mouse cardiomyocytes were determined by qRT-PCR (n = 6 and 7; SEM; * t-test p = 0.0469 for Sarrah TH region, p = 0.0469 for ITPR2, p = 0.0313 for OXCT1).

Supplementary Figure 8

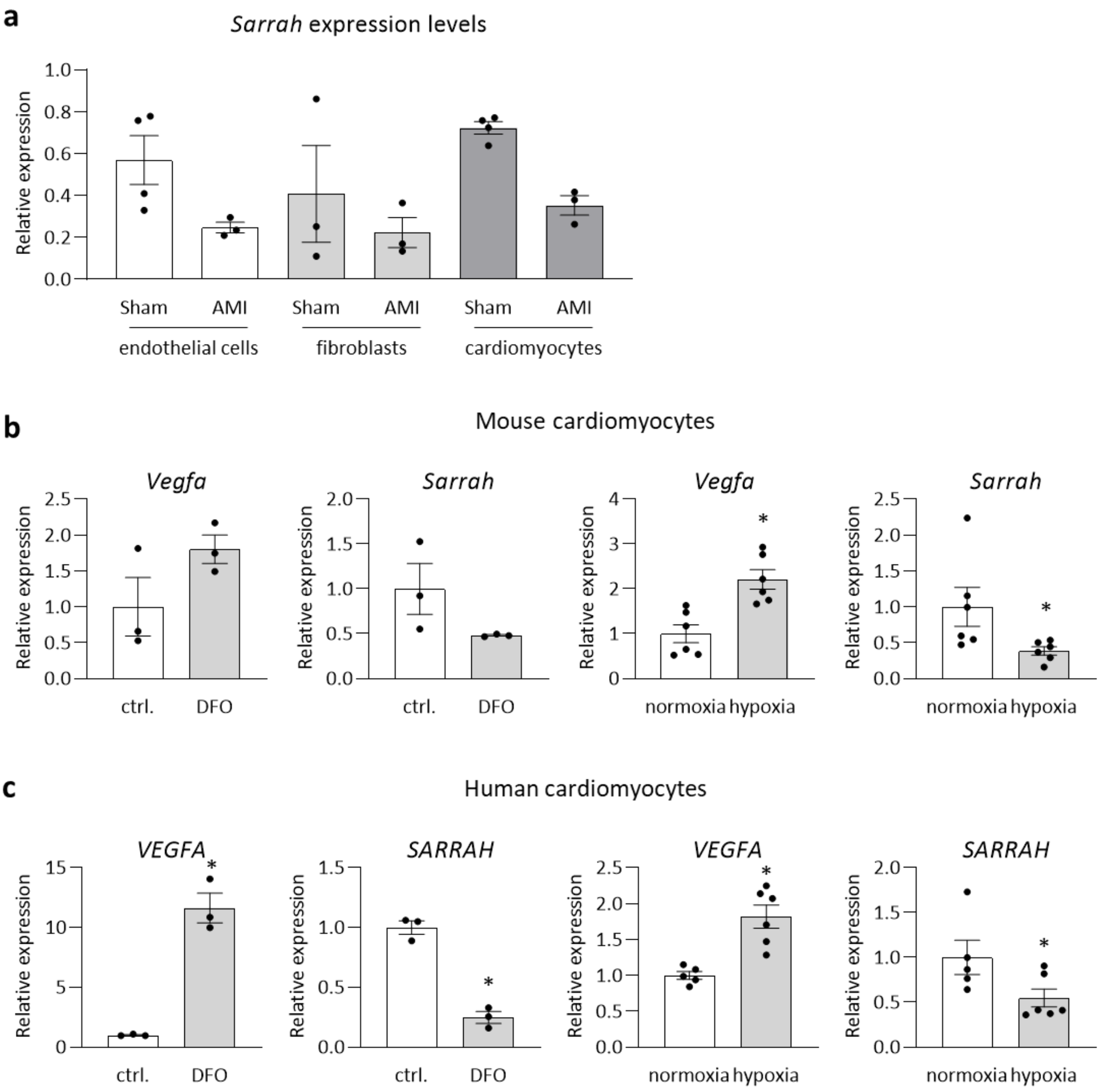

Supplementary figure 8 *Sarrah* is downregulated by hypoxia in different cardiac cell types in vivo and in cardiomyocytes in vitro.

a. *Sarrah* expression levels in endothelial cells, fibroblasts and cardiomyocytes isolated from mouse hearts 3 days after acute myocardial infarction, as measured by RNA sequencing by Rogg *et al.* (Circulation, 2018, 138 (22), 2545-2558) (n = 3 and 4; SEM).

b. Hypoxia was induced in mouse (HL-1 cell line) cardiomyocytes by treatment with 300 µM *deferoxamine mesylate* (DFO), a chelating compound, or by exposure to hypoxia (1 % O_2). VEGFA upregulation was measured by qRT-PCR as a hypoxic marker; *Sarrah* levels were measured by qRT-PCR (n = 3 and 6; SEM; * t-test p = 0.002 for Vegfa, p = 0.0087 for Sarrah).

c. Hypoxia was induced in primary human cardiomyocytes by treatment with 300 µM *deferoxamine mesylate* (DFO), a chelating compound, or by exposure to hypoxia (0.2 % O_2). VEGFA upregulation was measured by qRT-PCR as a hypoxic marker; *SARRAH* levels were measured by qRT-PCR (n = 3 and 6; SEM; * t-test p = 0.001 for VEGFA (with DFO), p = 0.0005 for SARRAH (with DFO), p = 0.004 for VEGFA (with hypoxia), p = 0.03 for SARRAH (with hypoxia)).

Supplementary Figure 9

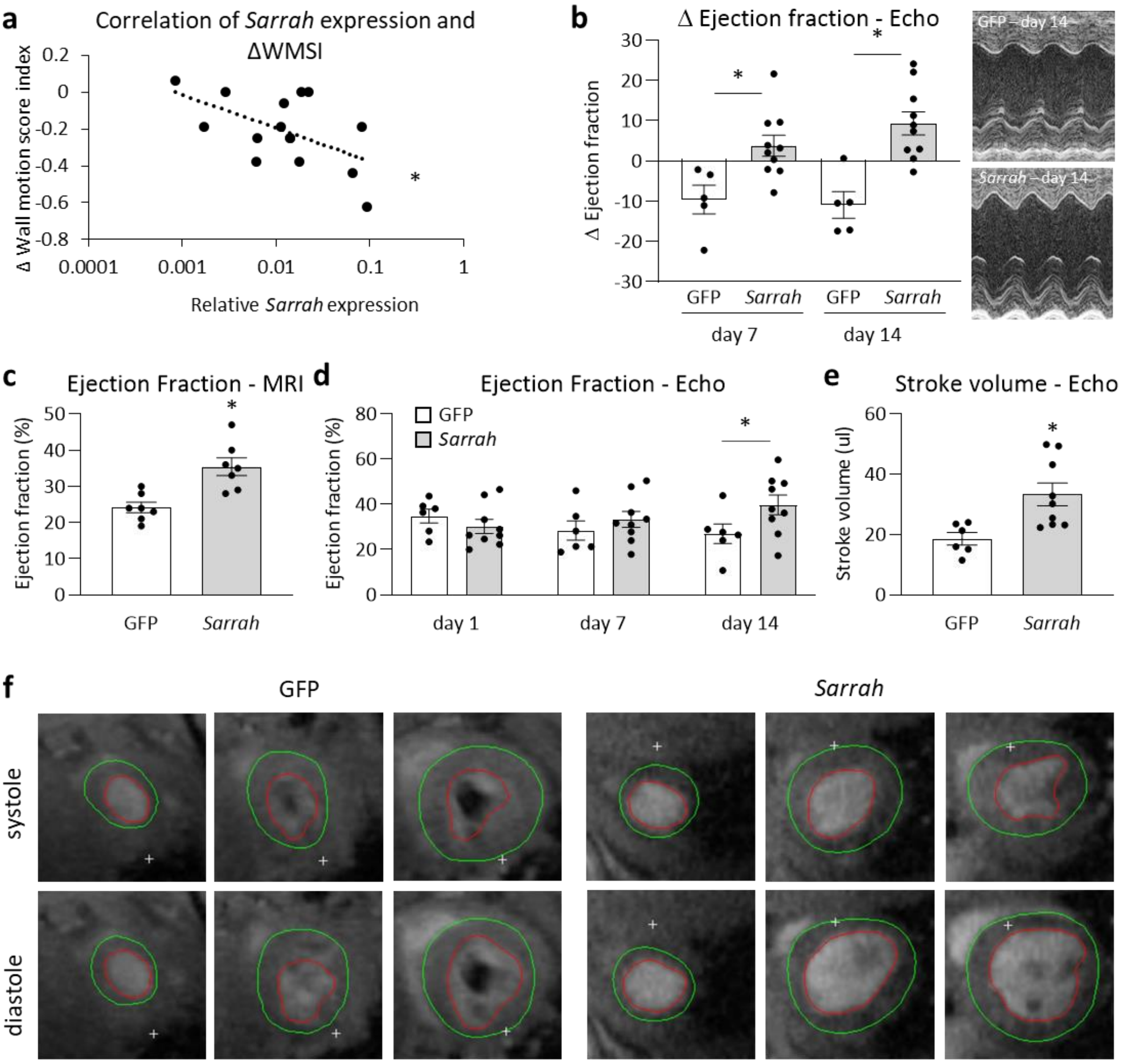

Supplementary figure 9 *Sarrah* overexpression in mice augments parameters of contractility after acute myocardial infarction.

a. Combined data from green fluorescent protein (GFP) and *Sarrah* overexpressing mice have been compared with regard to *Sarrah* expression levels at day 14 after AMI and changes in WMSI as assessed by echocardiography. The comparison shows the correlation between *Sarrah* levels and contractile function (n = 15; *Spearman correlation p = 0.0461).
b. Cardiac contractile function was assessed by echocardiography. Displayed are delta ejection fraction values on days 7 and 14 after AMI in comparison to day 1 and representative M mode echocardiography images on day 14 (AMI; n = 6 and 9; SEM; * t-test p = 0.0047 for day 7, p = 0.0027 for day 14).
c. Cardiac contractile function was assessed by magnetic resonance imaging (MRI). Displayed are ejection fraction values on day 14 after AMI (n = 7 and 8; SEM; * t-test p = 0.002).
d. Cardiac contractile function was assessed by echocardiography. Displayed are ejection fraction values on days 1, 7 and 14 after AMI (n = 6 and 9; SEM; * two-way ANOVA, F = 1.9 for variable treatment, p = 0.027).
e. Stroke volume was calculated as the difference between end diastolic and end systolic volumes, which were assessed from echocardiography at day 14 after AMI surgery (n = 6 and 9; SEM; * t-test p = 0.01).
f. Representative images from MRI on day 14 after AMI during end systole and end diastole from apical to basal heart segments.

Supplementary Figure 10

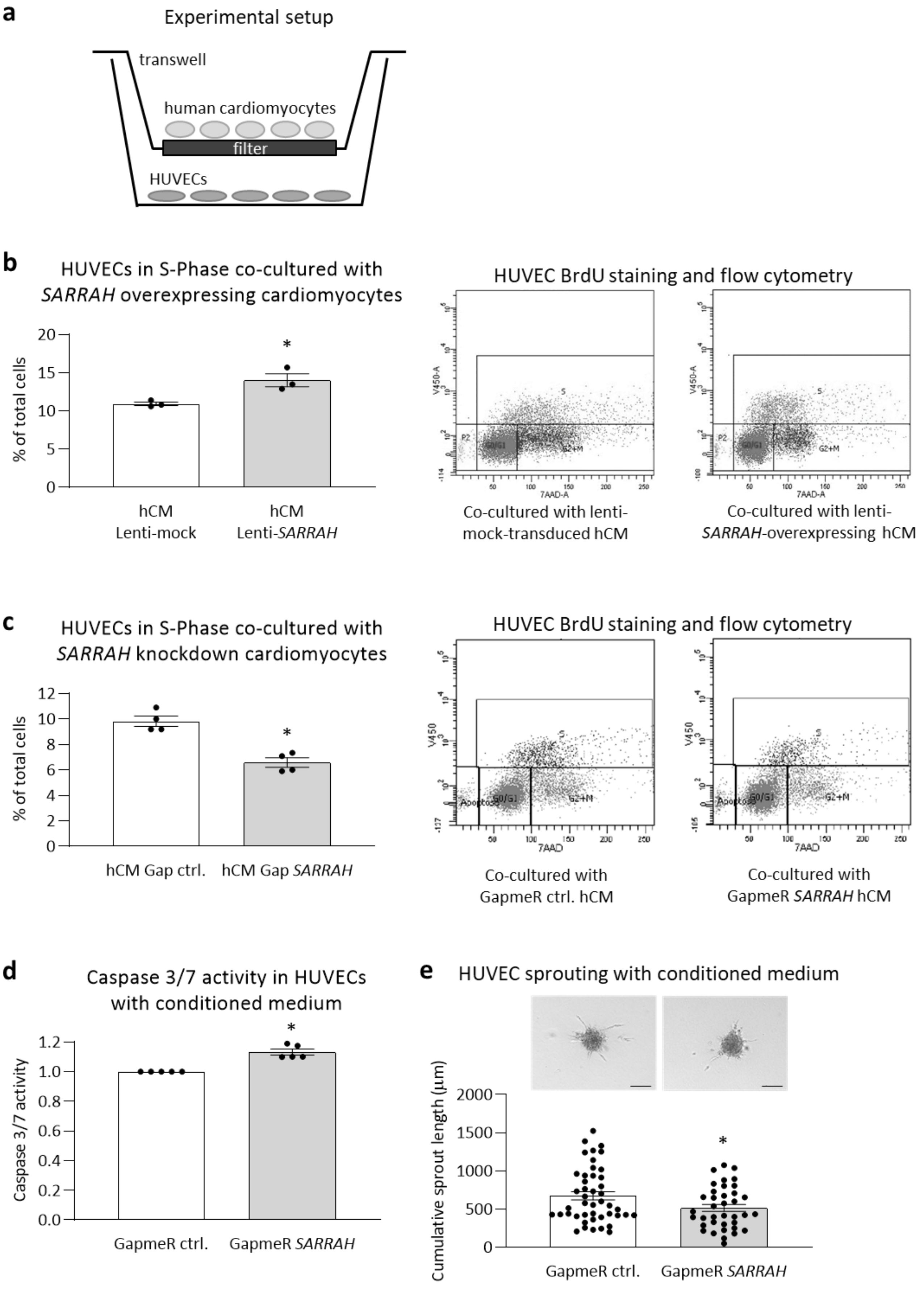

Supplementary figure 10 *SARRAH* promotes endothelial cell proliferation *in vitro*.

a. Scheme showing co-culture setup of transfected primary human cardiomyocytes and primary human umbilical vein endothelial cells *(*HUVECs).

b. Human cardiomyocytes were transduced with lentiviral vectors for overexpression and co-cultured with HUVECs on a transwell after medium change. Proliferation of HUVECs was assessed by bromodeoxyuridine (BrdU) staining and flow cytometry analysis after two days of co-culturing ($n = 3$; SEM; * t-test $p = 0.025$).

c. Human cardiomyocytes (hCM) were transfected with GapmeRs and co-cultured with HUVECs on a transwell after medium change. Proliferation of HUVECs was assessed by *bromodeoxyuridine* (BrdU) staining and flow cytometry analysis after two days of co-culturing ($n = 4$; SEM; * t-test $p = 0.01$).

d. Caspase-3/7 activity was measured in primary HUVECs cultured in conditioned medium from GapmeR-treated human cardiomyocytes ($n = 5$; SEM; * t-test $p < 0.003$).

e. Sprouting of primary HUVEC spheroids cultured in conditioned medium from GapmeR-treated human cardiomyocytes was assessed by cumulative sprout length ($n = 39$ and 46; SEM; * t-test $p = 0.03$; representative images are shown; scale bars are 100 µm).

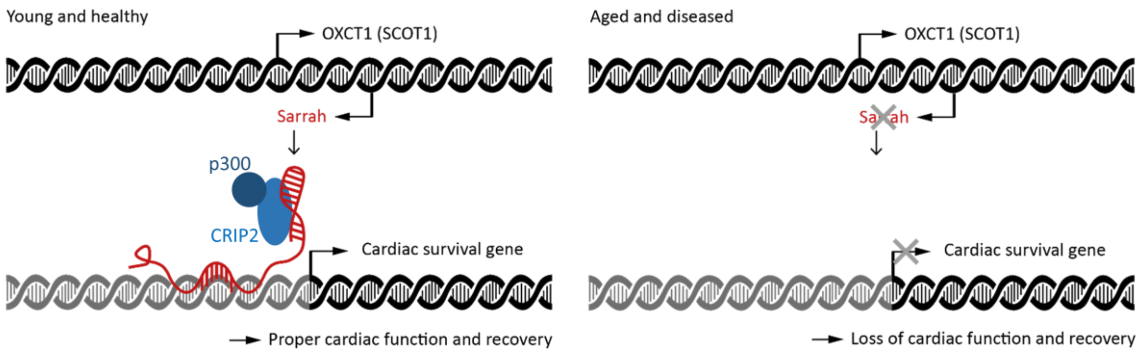

Supplementary figure 11 *SARRAH* forms triple helices within promoters of cardiac survival genes, such as NRF2, and activates expression via recruitment of CRIP2 and p300.

Scheme illustrating the proposed mechanism via which *Sarrah* acts: In young and healthy cardiomyocytes, *Sarrah* is sufficiently expressed and forms triple helices with gene promoters, thereby recruiting the transcripton factor CRIP2 and the transcriptional co-activator p300 and activating expression of cardiac survival genes, including NRF2. As a result, *Sarrah* contributes to proper cardiac function and recovery. In aged and diseased cardiomyocytes, *Sarrah* levels decrease entailing reduced expression of cardiac survival genes, a loss of cardiac function and the ability to recover.

Supplementary Figure 12

Example flow cytometry gating strategy for BrdU / 7-AAD proliferation analysis

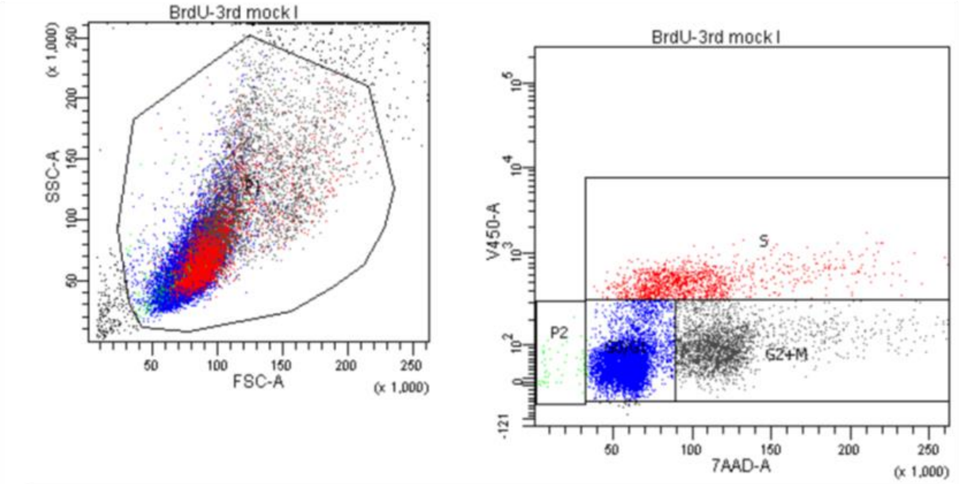

Experiment Name: Julia BrdU 20190301_gates
Specimen Name: BrdU
Tube Name: 3rd mock I
Record Date: Mar 1, 2019 2:20:26 PM
$OP: Boon

Population	#Events	%Parent	%Total	7AAD-A Mean	V450-A Mean
P1	19,940	96.5	96.5	78,902	125
G0/G1	12,780	64.1	61.9	59,179	57
G2+M	4,693	23.5	22.7	122,838	109
S	2,436	12.2	11.8	101,509	519
P2	128	0.6	0.6	18,538	40

Example flow cytometry gating strategy for Annexin V / 7-AAD apoptosis analysis

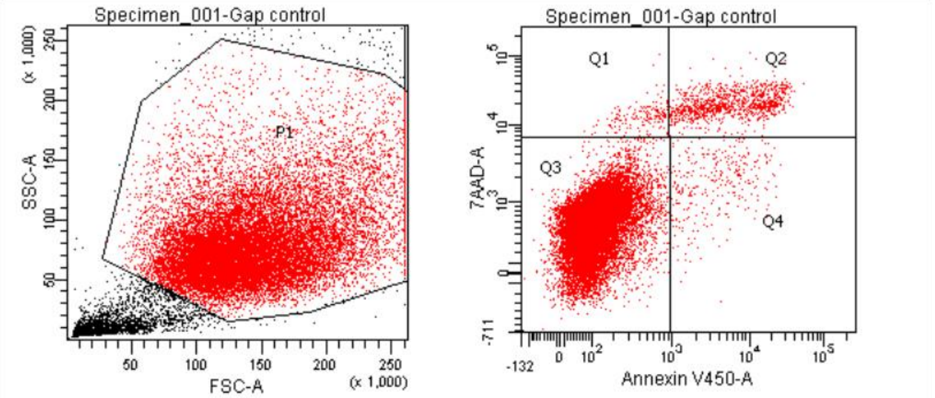

Experiment Name: 170316_Julia_ Annexin_HL1_24 h
Specimen Name: Specimen_001
Tube Name: Gap control
Record Date: Mar 17, 2016 12:48:45 PM
$OP: Julia

Population	Parent Name	#Events	%Parent	%Total	7AAD-A Mean	Annexin V45... Mean
All Events	####	23,823	####	100.00	1,839	792
P1	All Events	20,000	83.95	83.95	1,821	710
Q1	P1	161	0.80	0.68	14,023	494
Q2	P1	1,055	5.27	4.43	20,845	9,463
Q3	P1	18,480	92.40	77.57	624	132
Q4	P1	304	1.52	1.28	2,107	5,552
P2	P1	1,219	6.10	5.12	19,909	8,279
P3	P1	1,328	6.64	5.57	16,831	8,766

Supplementary Table 1

a

Species	*Sarrah* sequence ID
Human	OXCT1-AS1
Mouse	ENMUST00000140003
Rat	Not annotated
Pig	HX215121

b Coding potential of human *SARRAH*

Gene	RNA [nt]	ORF [nt]	Coding probability
SARRAH	1,731	300	0.05
MALAT1	8,779	213	0.01
XIST	19,275	411	0.03
OXCT1	3,388	1,563	1.00
FBXO4	1,682	1,164	1.00
c5orf51	5,241	885	0.99

c Coding potential of mouse *Sarrah*

Sequence	RNA [nt]	ORF [nt]	Coding probability
Sarrah	3,990	279	0.23
Malat1	8,779	213	0.07
Xist	17,918	519	0.04
Oxct1	3,496	1,563	1.00
Fbxo4	3,479	1,158	0.99
Plcxd3	1,905	966	0.99

Supplementary table 1 *Sarrah* conservation and coding potential.

a. Sequence IDs of *Sarrah* homologues in different species.

b. Supplementary table summarizing the coding potential of human *SARRAH* as assessed using the coding potential assessment tool based on alignment-free logistic regression model. MALAT1 and XIST are included as examples of know non-coding transcript, *cis* genes of *SARRAH* as protein-coding examples.

c. Supplementary table summarizing the coding potential of mouse *Sarrah* as assessed using the coding potential assessment tool based on alignment-free logistic regression model. MALAT1 and XIST are included as examples of know non-coding transcript, *cis* genes of *Sarrah* as protein-coding examples.

Supplementary Table 2

Disease Category	p-value	Benjamini
Chemdependency	$3.6*10^{-10}$	$6.4*10^{-9}$
Cardiovascular	$5.8*10^{-8}$	$5.2*10^{-7}$
Metabolic	$5.5*10^{-6}$	$3.3*10^{-5}$
Psych	0.0022	0.01
Immune	0.0027	0.0096
Developmental	0.0031	0.0091
Aging	0.0032	0.0083
Cancer	0.0096	0.021
Hematological	0.027	0.053
Unknown	0.075	0.13
Neurological	0.086	0.14
Pharmacogenic	0.094	0.14

Supplementary table 2 Disease categories affected by *SARRAH*-induced genes.
Gene ontology analysis of all 134 genes in whose promoters *SARRAH* is predicted to form triple helices using the Database for Annotation, Visualization and Integrated Discovery (DAVID) revealed disease categories from the Genetic Association Database affected by *SARRAH* silencing.

Supplementary Table 3

Species	*Sarrah* region	Sequence	p-value	Number of targets
Human	332-353	GCCCCCTTCTCTTCTCCCGCCT	0.0033	135
Mouse	1399-1432	GTGTCTGTCTCCTCTCCTCCTCGTCCTTGTCCAT	0.0012	165

Supplementary table 3 *Sarrah* triple helix domains

Sarrah triple helix domain positions and numbers of target genes in human and mouse.

Supplementary Table 4

a **Human**

Gene name	Pimer forward sequence	Primer reverse sequence
SARRAH	CCTGGACTGCGTTCACGTTT	CTGCAAGCCTTGTTGCTCAC
GAPDH	ATGGAAATCCCATCACCATCTT	CGCCCCACTTGATTTTGG
RPLP0	TCGACAATGGCAGCATCTAC	ATCCGTCTCCACAGACAAGG
VEGF	CCCTGATGAGATCGAGTACA	AGCAAGGCCCACAGGGATTT
OXCT1	GCAACAATGCAGGGGTTGAC	ATCCTCTCTGCAAGTGTGCC
FBXO4	GCCGGTACAGTGTGATTCCA	CAGCCTCTGTATCCTGGACTTTTA
c5orf51	GTGTGGTGAGACGAGTGGAA	AAGTGATCATCTTCACCGGCA
CHMP4C	AGAAAACCAGGCATGTCGTC	CCCAAGCTGCCAATTGTTTG
ZNF280A	TGAAGAGACCAGTGGCTTCA	GTACATGCTCCACCCCAACA
GPRC5A	GAAGCAGCACCAAGTTCACG	CTGTTGGAGTCCTGCACCTT
BEX1	GCTGGTGAATACTGTGTGCC	ATCCTTGCCTGTGGTTCTCC
SPINK1	TGCACCAAGATATATGACCCTGT	GTTCTCAGCAAGGCCCAGAT
GPC6	TCACTCGGCCTGACACTTTC	AACTCAAACTCCGTGGGACA
DPYD	GATGCCCCGTGTCAGAAGAG	AATGGGTCCCTCTTCAGTGG
PSD3	AGGAGAAAGCTAACGGAACACA	TCCGAGCCAAGAATCCACTT
KMT2C	AGAACCCAGCTGAAGGACTG	GTCCGTTTGCTTCGCTGTTT
NEK10	CTTTGGCCTGGCAAAGCAAA	CCCCATACGGCTCACTCTTC
PDE3A	GCAGACCCTTCTCTTCCACC	ACTCGTCTCAACAAGCCAGG
ITPR2	GCCAACCCTCCCAAGAAGTT	TGTGGTTCCCTTGTTTGGCT
PARP8	GGGGATGTGTTCAAGGCAAG	GCAGTCCTTCTCAGCTCTGG
SSBP2	TGGGATCTCTACTGTGCAGC	CTAGCACTGGACTGGGAGC
NRF2	CAGCGACGGAAAGAGTATGA	TGGGCAACCTGGGAGTAG
U4 snRNA	GCCAATGAGGTTTATCCGAGG	TCAAAAATTGCCAATGCCG
GPC6 promoter	TGTTGCTTCTGTGGTCTGGT	ATGGGTGGCAGAGCACAATC
PDE3A promoter	CTGAAGTAGGAAGAGACCCCG	CGGGCAGAAACGATCAGGAT
ITPR2 promoter	AGTAGGAAGAGACCCCGGAG	AAGGCTAGCACGCTCAAGTT
PARP8 promoter	GGCTTGAACCTGTCCTACCC	TCCACTGCAGCCTTTGATGT
SSBP2 promoter	TTCCACACACACACAGCTTT	TCTCGACCTCTCACTTTTGCT
GAPDH promoter	TGGTGTCAGGTTATGCTGGGCCAG	GTGGGATGGGAGGGTGCTGAACAC

b **Mouse**

Gene name	Pimer forward sequence	Primer reverse sequence
Sarrah	GCCCAATGGCTGATAGTGTT	GTGGAGACCCAGAGCAGAAG
Gapdh	CATCTGAGGGCCCACTGAAG	GTTGCTGTTGAAGTCGCAGG
Rplp0	GCGTCCTGGCATTGTCTGT	GAAGGCCTTGACCTTTTCAGTAA
Vegf	CACGACAGAAGGAGAGCAGA	GGCAGTAGCTTCGCTGGTAG
Oxct1	GGACGGCATGTACGCTAACT	TCCGCATCAGCTTCGTCTTT
Fbxo4	TCAGCCTACAGAGTGAGGGG	TTGCCATTCATGACGTCGGT
Cdh5	AGCGCAGCATCGGGTACT	TCGGAAGAATTGGCCTCTGT
Tnnt2	TGAAGAAGCCAAAGATGCTG	CCTGCTGGGCTTGGGTTT
Gpc6	GACAGTGGGCAGAGAGGTTG	AAGGTCTCACAGTGGGCAAG
Pde3a	TCCAAGCGCCTGAGAAGAAG	GGCAGAGGTGGTAGTTGTCC
Itpr2	CCTGACGGTGAACAAGAGGT	TGCAGCATCCAGTGACACTC
Parp8	GGGGAGGAGTCAAGGCAAAA	GCTGAGGGTGCTTGTGTAGT
Ssbp2	TGCCTGGAATGAACATGGGT	AGGAGACGCTGAGGAGTAGG
Sarrah TH region	CTGGAACCGGAGTCCCAAC	CGAGGAGGAGAGGAGACAGAC

c **Rat**

Gene name	Pimer forward sequence	Primer reverse sequence
Sarrah	TAGGGGAAGGCAGGCATTTG	AGAAGCTAGACAGGGAGGGG
Hprt1	CCTCCTCCGCCAGCTT	GTCATAACCTGGTTCATCATCACT

Supplementary table 4 qRT-PCR primer sequences used in the study.

a. Human primer sequences used in the study.

b. Mouse primer sequences used in the study.

c. Rat primer sequences used in the study.

All sequences in 5’ to 3’ orientation.

Supplementary Table 5

a

LNA GapmeR name	LNA GapmeR sequence
GapmeR negative control A	AACACGTCTATACGC
mouse GapmeR *Sarrah*	TTGGAAAGGTGAGCTG
human GapmeR *SARRAH*	GGTTGCATCTTTAGTA
rat GapmeR *Sarrah*	TGAAGGTCGCCAATCC

b

siRNA name	siRNA forward sequence	siRNA reverse sequence
siRNA firefly luciferase GL2	CGUACGCGGAAUACUUCGA[dT][dT]	UCGAAGUAUUCCGCGUACG[dT][dT]
si*Sarrah*_1 (mouse)	UUCUGCCGAAAGUAUCCAG[dT][dT]	CUGGAUACUUUCGGCAGAA[dT][dT]
si*Sarrah*_2 (mouse)	UCCCAAUGCUCCUUAUCUC[dT][dT]	GAGAUAAGGAGCAUUGGGA[dT][dT]
siOxct1 (mouse)	GAGUUUAACGGUCAGCACU[dT][dT]	AGUGCUGACCGUUAAACUC[dT][dT]

c

sgRNA name	sgRNA sequence #1	sgRNA sequence #2
sgRNA mock	TCAACCCCAGCGCACCGTTG	GCAATGCAATCGCAGGAGCA
sgRNA *Sarrah*ΔTH	TGTTGTATAATTCCCCTCAC	GAGTCCCAACAATTCCAGAA

d

NMR oligo name	NMR oligo sequence
human *SARRAH* TH	CCCCCUUCUCUUCUC
human GPC6 promoter_s	CACCTCCCTTCCCCC
human GPC6 promoter_as	GGGGGAAGGGAGGTG
mouse *Sarrah* TH	UGUCUCCUCUCCUCC
mouse GPC6 promoter_s	AGAAAGGAGGGGAGG
mouse GPC6 promoter_as	CCTCCCCTCCTTTCT

e

RNA pulldown probe name	RNA pulldown probe sequence
scrambled	mAmGmUmGmUmUmAmCmGmGmUmCmGmAmCmCmAmAmCmAmA-iSp9-rArCrGrArUrC-3deSBioTEG
Sarrah antisense #1	mAmGmGmAmGmAmUmCmUmUmUmAmUmCmAmGmUmGmAmCmC-iSp9-rArCrGrArUrC-3deSBioTEG
Sarrah antisense #2	mUmUmUmCmAmUmGmGmAmUmCmUmCmUmCmUmAmGmGmCmA-iSp9-rArCrGrArUrC-3deSBioTEG

Supplementary table 5 Oligo sequences used in the study.

a. LNA GapmeR sequences used in the study.

b. siRNA sequences used in the study.

c. sgRNA sequences used in the study for CRISPR/Cas9-mediated excision of the mouse *Sarrah* triple helix domain.

d. DNA and RNA oligos used in the study for nuclear magnetic resonance experiments.

e. RNA oligos used for RNA pulldown experiments in the study; m denotes methylated RNA nucleotides, r denotes unmethylated RNA nucleotides.

All sequences in 5’ to 3’ orientation.
