# Supplementary material for: Aging-regulated anti-apoptotic long non-coding RNA Sarrah augments recovery from acute myocardial infarction
Source: Nat Commun. 2020 Apr 27;11:2039. doi: 10.1038/s41467-020-15995-2 (PMC7184724; doi:10.1038/s41467-020-15995-2)
Supplement: Supplementary file 3 — Reporting Summary [file 41467_2020_15995_MOESM3_ESM.pdf]

nature research

Corresponding author(s): Reinier A. Boon

Last updated by author(s): Feb 4, 2020

Reporting Summary

Nature Research wishes to improve the reproducibility of the work that we publish. This form provides structure for consistency and transparency in reporting. For further information on Nature Research policies, see Authors & Referees and the Editorial Policy Checklist .

Statistics

For all statistical analyses, confirm that the following items are present in the figure legend, table legend, main text, or Methods section.

n/a	Confirmed	
☐	☒	The exact sample size (*n*) for each experimental group/condition, given as a discrete number and unit of measurement
☐	☒	A statement on whether measurements were taken from distinct samples or whether the same sample was measured repeatedly
☐	☒	The statistical test(s) used AND whether they are one- or two-sided<br>*Only common tests should be described solely by name; describe more complex techniques in the Methods section.*
☐	☒	A description of all covariates tested
☐	☒	A description of any assumptions or corrections, such as tests of normality and adjustment for multiple comparisons
☐	☒	A full description of the statistical parameters including central tendency (e.g. means) or other basic estimates (e.g. regression coefficient) AND variation (e.g. standard deviation) or associated estimates of uncertainty (e.g. confidence intervals)
☐	☒	For null hypothesis testing, the test statistic (e.g. *F*, *t*, *r*) with confidence intervals, effect sizes, degrees of freedom and *P* value noted<br>*Give P values as exact values whenever suitable.*
☒	☐	For Bayesian analysis, information on the choice of priors and Markov chain Monte Carlo settings
☒	☐	For hierarchical and complex designs, identification of the appropriate level for tests and full reporting of outcomes
☒	☐	Estimates of effect sizes (e.g. Cohen's *d*, Pearson's *r*), indicating how they were calculated

Our web collection on statistics for biologists contains articles on many of the points above.

Software and code

Policy information about availability of computer code

Data collection	Triplex domain Finder software (published by Kuo et al., Nucleic Acids Research 2019: Detection of RNA–DNA binding sites in long noncoding RNAs), FASTA Sequence Comparison software
Data analysis	Segment 1.8, Cufflinks 2.1, MaxQuant 1.6.1.0, Perseus 1.6.1.3, MS Excel 2013, GraphPad Prism 8, IonWizard, StepOne 2.3, Vevo LAB 1.7.0, AxioVision SE64 Rel. 4.9.1

For manuscripts utilizing custom algorithms or software that are central to the research but not yet described in published literature, software must be made available to editors/reviewers. We strongly encourage code deposition in a community repository (e.g. GitHub). See the Nature Research guidelines for submitting code & software for further information.

Data

Policy information about availability of data

All manuscripts must include a data availability statement. This statement should provide the following information, where applicable:

- Accession codes, unique identifiers, or web links for publicly available datasets
- A list of figures that have associated raw data
- A description of any restrictions on data availability

The sequences for OXCT1-AS1 and ENSMUST00000140003 are publicly available at sequencing and annotation database UCSC Genome Browser (http://genome.ucsc.edu/).
For gene set enrichment analysis and gene ontology analysis, the Database for Annotation, Visualization and Integrated Discovery (DAVID) 6.7 (https://david-d.ncifcrf.gov/) was used.
Data are deposited on the GEO repository under the accession number GSE145697. All other relevant data are available from the authors upon request. To improve the transparency and the reproducibility of results a reporting summary is provided.

Field-specific reporting

Please select the one below that is the best fit for your research. If you are not sure, read the appropriate sections before making your selection.

- [x] Life sciences
- [ ] Behavioural & social sciences
- [ ] Ecological, evolutionary & environmental sciences

For a reference copy of the document with all sections, see nature.com/documents/nr-reporting-summary-flat.pdf

Life sciences study design

All studies must disclose on these points even when the disclosure is negative.

Sample size	For in vitro experiments, sample sizes were chosen based on previous experiences and standards in the field. Samples sizes for animal experiments were determined using power calculation.
Data exclusions	Significant outliers were identified using Grubbs' outlier test and excluded from analysis to to detect outliers in a univariate data set assumed to be normally distributed. The exlusion criterion was pre-established.
Replication	All results were reproduced in at least 3 technically independent replicates.
Randomization	Mice were randomized before AAV9 treatment. For in vitro experiments, samples were randomized before the respective manipulation within each experiment.
Blinding	All researchers involved in animal experiments were blinded to the treatment during data collection and analysis.

Reporting for specific materials, systems and methods

We require information from authors about some types of materials, experimental systems and methods used in many studies. Here, indicate whether each material, system or method listed is relevant to your study. If you are not sure if a list item applies to your research, read the appropriate section before selecting a response.

Materials & experimental systems

n/a	Involved in the study	
[ ]	[x]	Antibodies
[ ]	[x]	Eukaryotic cell lines
[x]	[ ]	Palaeontology
[ ]	[x]	Animals and other organisms
[x]	[ ]	Human research participants
[x]	[ ]	Clinical data

Methods

n/a	Involved in the study	
[x]	[ ]	ChIP-seq
[ ]	[x]	Flow cytometry
[x]	[ ]	MRI-based neuroimaging

Antibodies

Antibodies used

Anti-rabbit IgG, Millipore #12-370
Anti-rabbit IgG, Diagenode #15410206 (for triplex-IP experiments)
Anti-mouse IgG, Santa Cruz #2025
Anti-total H3, abcam #1791
Anti-CRIP2, Novus Bio #NBP2-59094
Anti-EP300, active motif #61401
Anti-H3K27ac, abcam #4729
Anti-DNA-RNA Hybrid [S9.6] Antibody, Kerafast #ENH001
Anti-PH3 (Ser10), Merck #6570
Anti-CD31, Thermo Fisher Scientific #14-0311-82
Anti-BrdU-V450, BD Biosciences #560810
Anti-annexin V-V450, BD Biosciences #560506
HRP-conjugated secondary anti-rabbit antibody, Dako, #P0448

Validation

All antibodies are commercially available and validated by the manufacturers.

Eukaryotic cell lines

Policy information about cell lines

Cell line source(s)	HL-1 cells were purchased from Sigma-Aldrich, HEK293T cells were purchased from Invitrogen, primary HUVECs were purchased from Lonza, hiPSC were obtained by reprogramming of fibroblasts from a healthy donor with Yamanaka factors using the CytoTune-iPS Sendai Reprogramming Kit (Life Technologies).
Authentication	The cell lines were not authenticated.
Mycoplasma contamination	All cell lines were tested negative for mycoplasma contamination.
Commonly misidentified lines (See ICLAC register)	No commonly misidentified cell lines were used in the study.

Animals and other organisms

Policy information about studies involving animals; ARRIVE guidelines recommended for reporting animal research

Laboratory animals	mouse, C57BL/6, male, 8 weeks, 12 weeks or 18 months old<br>rat, Sprague Dawley, male and female, neonatal, one day old<br><br>All animals were obtained from Charles River. Mice were kept in individually ventilated cages (Tecniplast) at 12:12 hour-light/ dark cycles. Water and ssniff R/M-H complete feed (ssniff Spezialdiäten, Soest, Deutschland) were fed ad libitum.
Wild animals	The study did not involve wild animals.
Field-collected samples	The study did not involve samples collected from the field.
Ethics oversight	Regierungspräsidium Darmstadt, Hesse, Germany

Note that full information on the approval of the study protocol must also be provided in the manuscript.

Flow Cytometry

Plots

Confirm that:

- [x] The axis labels state the marker and fluorochrome used (e.g. CD4-FITC).
- [x] The axis scales are clearly visible. Include numbers along axes only for bottom left plot of group (a 'group' is an analysis of identical markers).
- [x] All plots are contour plots with outliers or pseudocolor plots.
- [x] A numerical value for number of cells or percentage (with statistics) is provided.

Methodology

Sample preparation	HL-1 cells were fixated and stained using the anti-V450 Annexin V antibody (BD Biosciences #560506).<br>HUVECs were not fixated and staining using the BrdU Flow Kit (BD Biosciences).
Instrument	BD FACSCanto II machine (BD Biosciences)
Software	BD FACSDiva™ software
Cell population abundance	Only cultured cells were used for staining followed by flow cytometry, cells were not sorted.
Gating strategy	Cells were selected by FSC and SSC to analyze single cells. Boundaries between "positive" and "negative" staining cell populations were defined at < 1 % (for negative staining). Supplementary figure 12 shows example gating strategies.

- [x] Tick this box to confirm that a figure exemplifying the gating strategy is provided in the Supplementary Information.
